# Efficacy of Topical Application, Leaf Residue or Soil Drench of Blastospores of *Isaria fumosorosea* for Citrus Root Weevil Management: Laboratory and Greenhouse Investigations

**DOI:** 10.3390/insects7040066

**Published:** 2016-11-22

**Authors:** Pasco B. Avery, Wayne B. Hunter, David G. Hall, Mark A. Jackson, Charles A. Powell

**Affiliations:** 1 Indian River Research and Education Center, Institute of Food and Agricultural Sciences, University of Florida, 2199 South Rock Road, Fort Pierce, FL 34945, USA; capowell@ufl.edu; 2 Subtropical Insect Research Unit, U.S. Horticultural Research Laboratory, Agricultural Research Service, USDA, 2001 South Rock Road, Ft. Pierce, FL 34945, USA; wayne.hunter@ars.usda.gov (W.B.H.); david.hall@ars.usda.gov (D.G.H.); 3 Crop Bioprotection Research Unit, National Center for Agricultural Utilization Research, Agricultural Research Service, USDA, Peoria, IL 61604, USA; mark.jackson@ars.usda.gov

**Keywords:** blastospores, citrus, *Diaprepes abbreviatus*, soil drench, *Isaria fumosorosea*, biological control

## Abstract

The efficacy of topical, leaf residue, and soil drench applications with *Isaria fumosorosea* blastospores (*Ifr* strain 3581) was assessed for the management of the citrus root weevil, *Diaprepes abbreviatus* (L.). Blastospores of *Ifr* were applied topically at a rate of 10^7^ blastospores mL^−1^ on both the larvae and adults, and each insect stage was incubated in rearing cups with artificial diet at 25 °C, either in the dark or in a growth chamber under a 16 h photophase for 2 weeks, respectively. Percent larval and adult mortality due to the infection of *Ifr* was assessed after 14 days as compared to untreated controls. Leaf residue assays were assessed by feeding the adults detached citrus leaves previously sprayed with *Ifr* (10^7^ blastospores mL^−1^) in Petri dish chambers and then incubating them at 25 °C for 2–3 weeks. Efficacy of the soil drench applications was assessed on five larvae feeding on the roots of a *Carrizo* hybrid citrus seedling ~8.5–10.5 cm below the sterile sand surface in a single 16 cm × 15.5 cm pot inside a second pot lined with plastic mesh to prevent escapees. Drench treatments per pot consisted of 100 mL of *Ifr* suspension (10^7^ blastospores mL^−1^), flushed with 400, 900, or 1400 mL of water compared to 500, 1000, and 1500 mL of water only for controls. The mean concentration of *Ifr* propagules as colony forming units per gram (CFUs g^−1^) that leached to different depths in the sand profile per treatment drench rate was also determined. Two weeks post-drenching of *Ifr* treatments, larvae were assessed for percent mortality, size differences, and effect of treatments in reducing feeding damage to the plant root biomass compared to the controls. Topical spray applications caused 13 and 19% mortality in larvae and adults after 7 days compared to none in the control after 14 days, respectively. Adults feeding on a single *Ifr* treated leaf for 24 h consumed less than the control, and resulted in 100% mortality 35 days post-treatment compared to 33% in the untreated control. Although offered fresh, untreated leaves after 24 h, only adults in the control group consumed them. *Ifr* CFUs g^−1^ were isolated 8.5–10.5 cm below the sand surface for the 1000 and 1500 mL drench rates only, resulting in 2%–4% larval mortality. For all the *Ifr* drench treatments, no differences were observed in percent larval mortality and size or the effect of treatments in reducing feeding damage to the plant root biomass compared to the controls. These results suggest that the foliar application of *Ifr* may be an efficient biocontrol strategy for managing adult populations of *D. abbreviatus*; potential alternative larval management strategies are discussed.

## 1. Introduction

*Diaprepes* citrus root weevil (DRW), *Diaprepes abbreviatus* (L.), (Coleoptera: Curculionidae) was first reported in 1964 on the mainland USA, in Apopka, FL [1], and has since become a key pest of citrus and ornamental plants throughout the southern USA. According to Duncan et al. [2], the most common root weevil species infesting citrus in order of geographical distribution in Florida is the DRW. Egg masses on leaves hatch, the DRW larvae fall to the ground and then burrow through the soil, feeding on roots which can cause girdling of the tree tap root. This feeding can result in death or more often secondary infection of the structural roots or root crown by *Phytopthora* spp*.* wherein mature citrus trees may die [3,4]. Due to the difficulty of reducing subterranean insect pests, the DRW has become a major concern of citrus growers in Florida as few effective and environmentally appropriate control measures are currently available to growers [2,5]. Also, growing concerns about the negative effects of chemical insecticides on workers, food supply, and the environment make microbial control of arthropod pests of tree fruit crops an attractive alternative [6,7,8,9,10,11].

Suppression of immigrant insect populations in the soil and on plant materials can be effectively achieved through applied biological control [12]. Fungal entomopathogens which are natural suppressors of insects are often chosen as microbial control agents of polyphagous insect pests [13] and are considered valuable control agents of Coleoptera [11,13,14,15,16,17,18,19]. Four entomopathogenic fungi, *Metarhizium anisopliae* (Metschnikoff) Sorokin (Hypocreales: Clavicipitaceae), *Beauveria bassiana* (Balsamo) Vuillemin (Hypocreales: Clavicipitaceae), *Purpureocillium lilacinum* (Thom) Luangsa-Ard, Houbraken, Hywel-Jones and Samson [20] (=*Paecilomyces lilacinus*) (Hypocreales: Ophiocordycipitaceae) and *Aspergillus ochraceous* Wilhelm (Eurotiales: Trichocomaceae) were isolated from DRW larvae in a survey conducted monthly from June 1979 through December 1980 in nine citrus groves and one ornamental nursery in central Florida [15]. Dolinski and Lacey [9] reported that the fungal entomopathogen, *B. bassiana* provided effective control of adult and neonate larvae of DRW when applied to the soil under citrus trees. Also, *B. bassiana* has been demonstrated to infect the adult banana root borer, *Cosmopolites sordidus* (Germar) (Coleoptera: Curculionidae), and West Indian sugarcane borers, *Metamasius hemipterus sericeus* Olivier (Coleoptera: Curculionidae) [16]. Infection of the red palm weevil*, Rhynchophorus ferrugineus* Olivier (Coleoptera: Curculionidae) by *B. bassiana* was determined successful through identification of conidiophores on the insect’s cuticle indicating the completion of the fungus’s life cycle within the insect [11]. Another fungal entomopathogen, *Isaria fumosorosea* (*Ifr*) Wise (Hypocreales: Cordycipitaceae) has also been demonstrated to infect the Asian citrus psyllid, *Diaphorina citri* Kuwayama (Hemiptera: Liviidae), the insect vector of citrus greening disease [21,22,23,24,25,26,27,28] and other citrus pests, including the brown citrus aphid, *Toxoptera citricida* (Kirkaldy) (Hemiptera: Aphididae), glassy-winged sharpshooter, *Homalodisca vitripennis* (Germar) (Hemiptera: Cicadellidae), and DRW [29].

Therefore, to meet the industry needs, we evaluated the efficacy of application of *Ifr* for management of the DRW active life stages. Our interests were in finding effective, environmentally friendly, fungal entomopathogenic organisms which may be used as biological control agents against appropriate DRW stages in an Integrated Pest Management (IPM) agroecosystem program. Indeed, entomopathogenic fungi have been shown to be functional in managing pest populations when incorporated as part of an IPM program [10,30]. In addition, as part of an IPM strategy, the purpose of this study was to determine under laboratory and greenhouse conditions the efficacy of different application practices using *Ifr* for DRW management: topical application, leaf spray and soil drench.

## 2. Materials and Methods

### 2.1. Citrus Leaves and Plants

Duncan grapefruit (*Citrus paradisi* Macf.) seedlings were grown in Premier Pro-mix General Purpose Growing Medium from seed in size C10 “Cone-tainers”™ (Stuewe & Sons, Inc., Corvalis, OR, USA) for approximately 6 months. Detached leaves of similar age and size (1944 ± 42.9 mm^2^) were used for leaf residue bioassays.

Carrizo citrange trifoliate hybrid seedlings (*Citrus sinensis* ‘Washington’ sweet orange × *Poncirus trifoliata*) were grown the same as above for 6 months and then transplanted into 16 cm × 15.5 cm pots with sterile sand. Both detached leaves and potted plants used for topical and soil drench experiments were grown and obtained from the United States Department of Agriculture (USDA), Agricultural Research Service (ARS), United States Horticultural Research Laboratory (USHRL), in Fort Pierce, Florida.

### 2.2. Insects

DRW larval and adult life stages were obtained from an artificial colony maintained at the USDA, ARS, USHRL, in Fort Pierce, Florida and reared as described by Lapointe and Shapiro [5]. Both life stages were reared in Dart™ PC clear plastic cups (30 mL) with lids (Dart Container Corp., Mason, MI, USA) containing ~10 mL of artificial diet [5] until needed for experiments.

### 2.3. Fungal Blastospore Preparation and Viability

*Ifr* strain 3581 originally stored at the USDA, ARS, Collection of Entomopathogenic Fungal Cultures (ARSEF) was formulated with diatomaceous earth as dry powders in vacuum packed 10 g bags and obtained from the USDA, ARS, National Center for Agricultural Utilization Research, Crop Bioprotection Research Unit, in Peoria, IL. These dried blastospore formulations were produced and stabilized using methods previously described by Jackson et al. [31]. All dried blastospore formulations were stored at 4 °C prior to use. The blastospore suspension was prepared by mixing 2 g of the dried blastospore powder in 100 mL of sterile distilled water, stirring the suspension with a magnetic bar for 30 min, and allowing the diatomaceous earth to settle from the suspension. The supernatant of the blastospore suspension (50 mL) was pipetted into a Nalgene^®^ aerosol sprayer (Nalge Nunc International, Rochester, NY, USA). Two aliquots of the supernatant were taken from the suspension prior to spraying and the concentration of *Ifr* blastospores was determined using an improved Neubauer Bright-Line™ hemacytometer (Hausser Scientific, Horsham, PA, USA). The resulting concentration in the supernatant was 6.0 × 10^7^ blastospores mL^−1^.

To determine the viability of the blastospores, two potato dextrose agar (PDA: 3.9% w/v, Difco Laboratories, Detroit, MI, USA) Fisherbrand™ plates (100 mm × 15 mm; Thermo Fisher Scientific Co., Waltham, MA, USA) were sprayed. The plates were incubated for 12 h at 25 ± 1.0 °C and the percent viability was determined by microscopically viewing a total of 200 blastospores and determining if the spores had germinated. Blastospores were considered to have germinated if a germ tube had formed. Each plate was divided into 4 sections and 25 blastospores were observed (400×) in each of the four different sections per plate using a compound light microscope. This procedure was repeated for each experiment, and the mean percent viability of *Ifr* for all studies was ≥93%.

### 2.4. Topical Spray Application

**Figure 1:**
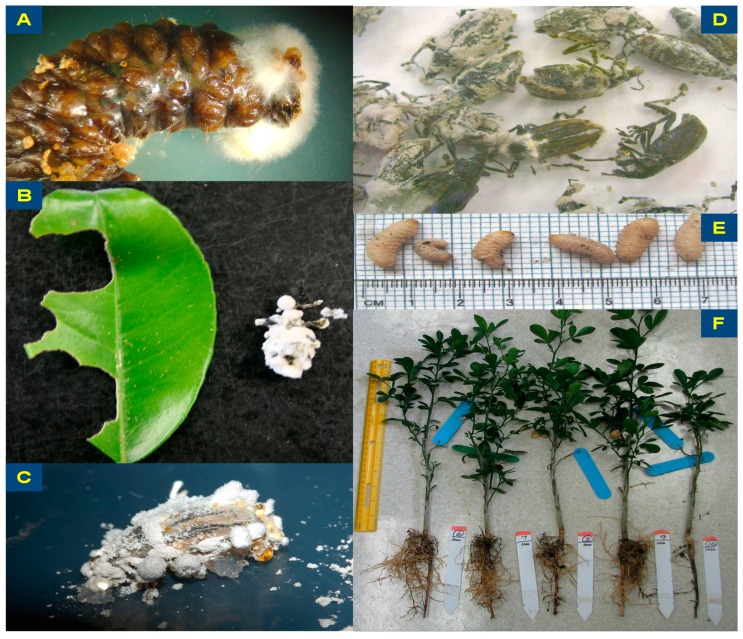
The photos are *Diaprepes* citrus root weevil (DRW) larvae and adults after infection due to *Isaria fumosorosea* (Ifr) as well as its effects on their growth and feeding behavior under different experimental conditions. (**A**) DRW larva mycosed with *Ifr*; (**B**) Duncan grapefruit leaf sprayed with *Ifr* and partially eaten by a DRW resulting in infection and colonization of adult beetle; (**C**) Colonized DRW beetle with *Ifr* after being incubated and then removed from a water agar plate; (**D**) DRW adult beetles colonized with *Ifr* after more than 30 days post-infection with conidia formed on the exoskeleton; (**E**) DRW larvae post-drench of *Ifr* showed no significant differences in size or average mass compared to the control; (**F**) Biomass of citrus seedlings showed no significant differences between *Ifr* drench treatments in growth or root feeding by DRW larvae assessed post treatment compared to the control.

Blastospores of *Ifr* or water was applied on 7 or 8 DRW 3rd–4th instar larvae or 2–3 day old adults (male and female). Adults were chilled in the refrigerator at 4 °C for 2–3 min prior to spraying. Groups of 4–5 of the same life stage were randomly placed in a square Falcon^®^ Petri dish (100 mm × 15 mm; Corning Inc., Corning, NY, USA) spraying platform containing moistened Fisherbrand™ filter paper circle (7.0 cm diameter; Thermo Fisher Scientific Co., Waltham, MA, USA) along with 2 plastic Fisherbrand™ coverslips (22 mm × 22 mm; Thermo Fisher Scientific Co., Waltham, MA, USA) randomly placed to determine spray deposition of blastospores mm^−2^. The Petri dish platform was held at ~45° angle and each group was sprayed with a Nalgene^®^ aerosol sprayer with either sterile distilled water (control) or an *Ifr* blastospore suspension (6.0 × 10^7^ blastospores mL^−1^) in sterile distilled water. The larval groups were then allowed to air dry for 30 min in a fume hood and adults were allowed to dry in a separate covered Handi-Foil™ Eco-Foil™ medium roaster pan (40.1 cm × 28.7 cm × 7.3 cm; Handi-foil Bake America, Wheeling, IL, USA). All plastic coverslips were removed from the spray platforms and allowed to air dry until assessed. Each group of the same DRW life stage once dry, were individually placed in a clear plastic rearing cup filled with artificial diet as described in Lapointe and Shapiro [5]. The snap cap lid with several pin holes for ventilation was replaced and then the cups with the insect life stage in a tray were incubated at 25 °C, either in the dark in a lab drawer for the larvae or in a growth chamber under a 16 h light: 8 h dark photoperiod for adults. Mortality due to fungal infection was assessed daily for 2 weeks post-treatment. Dead beetle life stages were removed from their rearing cups, placed on Petri dish plates containing water agar, sealed with Parafilm™ (Bemis Company, Inc., Neena, WI, USA) and transferred to the growth chamber in order to confirm the *Ifr* fungal phenotype (Figure 1A–D). Mean spray deposition of *Ifr* viable blastospores mm^−2^ per life stage was determined using the technique described by Avery et al. [22]. This experiment was conducted twice.

### 2.5. Detached Leaf Bioassay

Petri dishes (100 mm × 15 mm) which contained filter paper (7.0 cm diameter) moistened with 800 µL of sterile distilled water were used to house the adult beetles. Similar size Duncan grapefruit leaves (972 ± 21 mm^2^) were surface sterilized with a 2% bleach solution, rinsed 3 times in sterile distilled water and allowed to air dry. Once dry, they were placed on moistened filter paper in a separate Petri dish spraying platform along with 2 plastic coverslips used for determining spray deposition of spores. The Petri dish platform (same as described above) was held at ~45° angle and each leaf was sprayed on the adaxial side only with a Nalgene^®^ aerosol sprayer until runoff with either sterile distilled water or an *Ifr* blastospore suspension (6.0 × 10^7^ blastospores mL^−1^) in sterile water. Both the leaf and cover slips were removed, placed on brown paper towels in the fume hood, and allowed to air dry for 30 min. Dry leaves and plastic coverslips were removed, placed in clean Petri dishes and covered with lids until needed or assessed, respectively.

Fourteen adult beetles (male and female) were fed individually either a detached treated or untreated citrus leaf placed in a Petri dish. Dishes were held closed with 2 rubber bands (crisscross pattern) and then transferred to a growth chamber maintained at 25 °C under a 16 h photophase. Hereafter, these closed Petri dishes containing the leaf and adult DRW will be referred to as bioassay chambers. After 24 h, the original leaf was removed and assessed for area consumed and a new fresh untreated leaf was given to each adult/treatment as needed until death occurred. Assessment for leaf area consumed by adults consisted of tracing detached leaves on graph paper (1 mm^2^) before feeding DRW adults and subtracting the remaining leaf area from the original after feeding for 24 h. Dead beetles were removed from their bioassay chamber, placed on Petri dish plates containing water agar, sealed with Parafilm™ and transferred to the growth chamber in order to confirm the *Ifr* fungal phenotype (Figure 1A–D). Area of treated or untreated leaf consumed after 24 h and mortality due to fungal infection up to 5 weeks post-treatment was assessed. Mean spray deposition of *Ifr* viable blastospores mm^−2^ per leaf was determined the same as described above. All laboratory experiments were conducted twice.

### 2.6. Soil Drench Experiment

Soil drench experiments were conducted in the greenhouse (12–32 °C; 61–89% relative humidity) on DRW larvae feeding on the roots of a *Carrizo* citrange trifoliate hybrid citrus seedling ~8.5–10.5 cm below the sterile sand surface in a single black plastic gallon pot. Two weeks prior to this study, the potted seedling was placed inside of a second gallon plastic pot lined with plastic mesh, in order to prevent larvae from escaping out the bottom of the drainage holes. In each pot, 5 larvae of similar size and mass were recorded, released and allowed to burrow into the sand to gain access to the roots for feeding. The sand in each pot was saturated with water prior to the beginning of each experiment and allowed five days to insure that moisture was consistent per treatment. Each potted citrus seedling with larvae was drenched with 100 mL of *Ifr* suspension (10^7^ blastospores mL^−1^), and then flushed with either 400, 900, or 1400 mL of water per treatment. Control pots were drenched with 500, 1000, and 1500 mL of water per treatment. After two weeks post-drenching, larvae were sifted from the sand and held in Costar^®^12 well cell culture plates (Corning Inc., Corning, NY, USA) for assessment. The mass of both living and dead DRW larvae were recorded individually per group per *Ifr* treatment drench compared to water only. Dead larvae were washed gently with water, assessed and then the *Ifr* fungal phenotype was confirmed using the same protocol as described above for adults. Plants were also removed from the sand and roots washed with water. Mean plant biomass (wet weight after feeding root damage) and length of the longest seedling roots, was assessed compared to the control.

To determine the mean concentration of *Ifr* blastospores at different depths in the sand was able to leach per treatment drench rate, a technique modified from McCoy et al. [32] was employed. Pots were prepared the same as described above except without a citrus seedling and larvae added. Immediately after flushing *Ifr* suspensions at the above rates in 15 additional pots (5 pots/treatment rate), plastic graduated Falcon^®^ 25 mL (Corning Inc., Corning, NY, USA) sterile plastic disposable pipettes with the tips sawed off at each 3 mL mark were used for sand core sampling. Two modified pipettes randomly placed per pot were pushed into the moist sand to a maximum depth of 10.5 cm, removed gently and the tip sealed with Parafilm™, placed in a plastic container and stored horizontally at 4 °C in darkness until assessed. The quantification and depth of *Ifr* propagules as colony forming units (CFUs) for each moist sand core sample collected in the pipette was then assessed using the dilution plating method described by Goettel and Ingilis [33]. Each modified pipette from the same treatment rate was secured with a bench clamp to the laboratory bench so that the pipette extended beyond the edge and then 3 mL (=2.1 cm) sections were removed using a hack saw. As each inoculated sand section per depth collected in the pipette was being cut, some of the sand which emptied was collected in a clean plastic Petri dish bottom held underneath while cutting. Once the entire section was cut, the remaining sand was also placed in the dish bottom and all the sand per section was then emptied into a re-sealable plastic bag. Each bag containing a total of 10 sand core samples from all the sections at the same depth per treatment rate was sealed and thoroughly mixed by shaking and stored at 4 °C until used. Ten grams of sand from each bag were placed into a sterile plastic 50 mL centrifuge tube with 30 mL of sterile distilled water, thoroughly shaken by hand and then vortexed for 30 s. After the sand precipitated out of the solution, then 1 mL of the supernatant was serial diluted (10^−1^) and 50 µL of the 10^−1^ dilution was spread onto 5 individual PDA plates with streptomycin (0.01% v/v) and Dodine fungicide (0.02% v/v) added for a total of 25 plates/treatment for each depth assessment. In addition, the leachate from the bottom of 3 pots/treatment dilution was collected in a beaker and aliquots (4 µL) of the combined leachate from 3 pots/treatment were spread on 3 individual PDA plates with streptomycin and Dodine added to determine if any spores were present. Plates were then sealed with Parafilm and transferred to the growth chamber held at 25 °C with a 16 h photophase for 7–14 days until *Ifr* CFUs could be identified and quantified. All experiments were repeated on separate occasions.

### 2.7. Statistical Analysis

Significance for percent mortality of the DRW larval and adult stages after being sprayed with *Ifr* were compared and assessed using an independent samples *t*-test (α = 0.05) because there was no mortality in the controls. Significance in leaf area consumed by the adult beetles (male and female) per treatment were compared using an independent samples *t*-test (α = 0.05), and the resulting median survival time in days was compared using the Kaplan-Meier survival analysis (α = 0.05) (JMPPRO 12, Inc. for Windows 2016) (SAS Institute Inc., Cary, NC, USA). For the soil drench experiment, the percent mortality, plant biomass, seedling root length and quantification of CFUs per depth or per dilution rate were assessed using a one-way ANOVA. If treatment means were significantly different, then they were separated using a Tukey’s HSD test (α = 0.05). All analyses using PROC TTEST, PROC GLM were conducted with the SAS 9.4 program for Windows (2002–2012, SAS Institute Inc., Cary, NC, USA).

## 3. Results

### 3.1. Topical Spray Application

The mean ± SE for spray deposition of *Ifr* was 505 ± 133.1 viable blastospores mm^−2^ for both DRW larvae and adults. Mean percent mortality for topical spray applications of *Ifr* on larvae (13% ± 8.5%) and adults (19% ± 10.1%) after 14 days was similar (*t* = 0.47; df = 1, 30; *p* = 0.64), respectively (Figure 1A,D). None of the larvae or adults died in the control for either trial.

### 3.2. Detached Leaf Bioassay

The mean ± SE for *Ifr* deposition on the leaves sprayed to runoff per treatment was 1737 ± 570.8 viable blastospores mm^−2^. When DRW adults were fed only one *Ifr* treated leaf, 100% mortality occurred compared to 33% in the control 35 days post-treatment (Figure 1B–D).

Kaplan-Meier analysis (censored at 35 days) revealed that adults, after feeding on citrus leaves sprayed with *Ifr*, survived for a significantly shorter time period compared to those in treatments sprayed with water only (control), i.e., average survival ± SE was 18 ± 2.1 days versus 31 ± 2.2 days, respectively (log rank *χ*^2^ = 15.8, *p* < 0.0001, df = 1). Natural mortality in the control (13.3%) did not start until after 15 days post-feeding, when the treated adults were 3.5 times higher at 46.7% for the same duration. There were no significant differences in leaf area consumed for the first 24 h between male and female DRW adults for either the *Ifr* treatment (*t* = −0.08; df = 1, 26; *p* < 0.9372) or control (*t* = −1.42; df = 1, 26; *p* < 0.1662). However, the mean leaf area consumed by adults for the first 24 h in the *Ifr* treatment (720 ± 35.8) was significantly lower (*t* = 2.80; df = 1, 54; *p* < 0.0070) compared to the control (869 ± 39.7). After 24 h, only adults previously exposed to the "control" treatment consumed the new untreated fresh leaves.

### 3.3. Soil Drench Experiment

**Figure 2:**
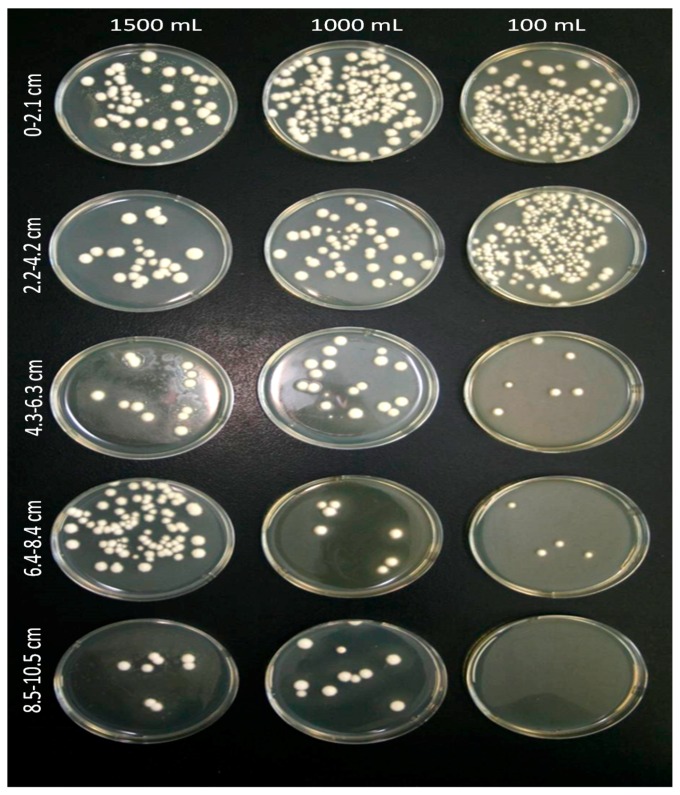
A visual depiction of the sand profile showing proportion of colony forming units (CFUs) isolated at different depths in pots drenched with 500, 1000 and 1500 mL of water that were previously inoculated with *Isaria fumosorosea*. The horizontal axis is the total amount of water used for drenching; vertical axis is the depth of the sand from the surface in each pot.

All the soil drench application rates of 400, 900, and 1400 mL of water plus 100 mL of *Ifr* suspension resulted in 2–4% DRW larval mortality, but the mortality was not significantly (*F* = 0.63; df = 5, 40; *p* < 0.6771) different compared to the control. However, both the 1000 and 1500 mL drench treatments allowed the *Ifr* blastospores to leach 8.5–10.5 cm below the sand surface compared to only 6.4–8.4 cm for the 500 mL treatment (Figure 2). Only in the 1500 mL drench treatments was there any leachate water runoff collected from the bottom of the pots; however, no *Ifr* CFUs were present.

**Figure 3:**
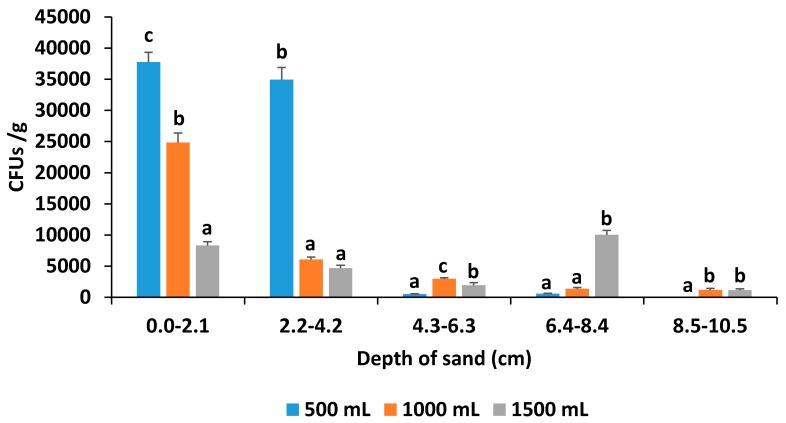
Comparison of the mean number ± SE of colony forming units per gram (CFUs/g) isolated at different depths of the sand profile in pots drenched with 500, 1000 and 1500 mL of water that were previously inoculated with *Isaria fumosorosea.* Letters above the bars per depth that are not the same are significantly different (Tukey’s HSD test, *p* < 0.05).

The number of *Ifr* CFUs per depth of the sand profile varied significantly amongst the different drench treatment rates (Figure 3). The highest (*F* = 108.4; df = 2, 18; *p* < 0.0001) number of *Ifr* CFUs/g (37,770) from 0 to 2.1 cm below the surface was found for the lowest drench rate of 500 mL, followed by 1000 mL (24855) and then 1500 mL (8310). At the depth of 2.2–4.2 cm below the surface, the mean number of CFUs/g (34,950) for the 500 mL drench rate was significantly higher (*F* = 180.2; df = 2, 18; *p* < 0.0001) compared to the other rates; however, both the middle and highest rates were similar. For the middle drench rate of 1000 mL, the mean number of CFUs/g (2985) were significantly higher (*F* = 26.4; df = 2, 18; *p* < 0.0001) compared to the highest rate (1920) which was also significantly different from the lowest rate (495). The mean number of CFUs/g (10,020) isolated from 6.4–8.4 cm below the surface was significantly higher (*F* = 164.3; df = 2, 18; *p* < 0.0001) compared to the other rates which were both similar. A similar number of CFUs/g (1200 and 1155, respectively) for both 1000 and 1500 mL were isolated at a depth of 8.5–10.5 cm below the surface, but none were found for the lowest drench rate.

At the end of the 14 day experiments after the sand was sifted, the DRW larvae in pots drenched with *Ifr* at different rates did not show any significant differences (*F* = 1.45; df = 5, 180; *p* = 0.2089) in average mass, compared to the control for each rate (Figure 1E). The mean percent mortality was about 2%–4% per pot for all *Ifr* drench treatments, but was not significantly different from the control (*F* = 0.63; df = 5, 40; *p* = 0.6771). Also, the biomass of citrus seedlings assessed post-drenching showed no significant differences (*F* = 1.28; df = 5, 45; *p* = 0.2902) amongst the *Ifr* drench treatments in growth or the effect of treatments in reducing feeding damage to the plant roots compared to the controls (Figure 1F).

## 4. Discussion

Based on the results of these experiments, DWR is susceptible to infection by blastospores of *Ifr* but the mortality varied depending on the type of inoculation and the life stage of the insect. In addition, the variation in mortality may be due in part to the effectiveness of the species-specific immunity of each individual host insect to resist the *Ifr* infection process [13]. In this study, the percent mortality for DRW larvae and adults when *Ifr* was topically applied reached only 13% and 19%, respectively. The low mortality using different entomopathogenic fungi against DRW is corroborated by other studies and was also observed in surveys taken in citrus groves throughout Florida [14,15]. In the laboratory, the fungus *M. anisopliae* infected 6.7% of the adults and none of the larvae. However in contrast, *B. bassiana* infected 92.7% of the adults within 7 days and 76.9% of the larvae within 12 days [14].

Other reasons for low larval mortality due to infection by *Ifr* could be attributed to the behavior of the beetle larvae burrowing into the artificial medium and physically removing some of the inoculum [34]. Dislodging of the spores after inoculation by the larval DRW burrowing into sand or soil has been observed [35]. However, in adults, it could be that the *Ifr* blastospores may become dislodged by not attaching securely due to the sclerotized hydrophobic elytra, thereby reducing the inoculum and potential pathogenicity of the entomopathogenic fungi. For example, in a study where DRW adults were inoculated with *B. bassiana* spores, then washed off and plated, the authors found that the spore density on the weevils’ surface was 15,815 spores initially, but 3 h after inoculation was reduced by 3 fold to 5782 spores [36]. The authors attributed the reduction to weevil activity, contact, or self-grooming observed during the study. In addition, another of their research findings concurred with ours, in that we also observed spores tended to condense in intersegmental areas and around hairs and the base of appendages including natural grooves. Also, due to the hydrophilic surfaces of the in vitro *Ifr* blastospores, they will not bind strongly with hydrophobic surfaces found on adult beetle surfaces and thereby may be removed more easily [37]. Factors that may inhibit or enhance germination and penetration include cuticle density or compounds on the insect integument [38,39]. In addition, the lack of nutrients on sclerotized beetle cuticle is a limiting factor in fungal growth and development [40]. Other physical or chemical aspects defining the interactions at the cuticle barrier between the entomopathogenic fungus and insect that ultimately can lead either to successful mycosis by the entomopathogen or successful defense by the host have been thoroughly reviewed [41].

The control technique that was most successful, resulting in 100% mortality, was after the DRW adults fed on a single leaf sprayed with *Ifr* blastospores. Having the blastospores gain entry in to the DRW adult by the mouth *per os* enhances the efficacy of the pathogen to enter the hemocoel faster, than via penetration through the cuticle [42]. Also, the DRW adults when presented the contaminated leaf sprayed with *Ifr* consumed significantly less than those presented with water treated leaves for the first 24 h, and thereafter only the adults in the control group consumed more leaves. This anti-feedant effect and subsequent behavior has been observed with other beetles after feeding on host plant leaves contaminated with fungal entomopathogens. For example, a reduction in feeding on potato leaves was observed for the Colorado potato beetle, *Leptinotarsa decimlineata* Say, infected by *B. bassiana* [43]. Consumption by the cowpea leaf beetle, *Ootheca mutabilis* Shalberg, treated with *B. bassiana* CPD 3 in feeding assays was significantly reduced within 2 days post-treatment, and this trend continued until 7 days when all the beetles died [44]. Most recently, this trend was also observed where the yellowmargined leaf beetle, *Microtheca ochroloma* Stål, consumption of bok choy leaves treated with *Ifr* was significantly reduced compared to the untreated plants [45].

This study is the first to investigate and determine both the ability and depth that *Ifr* blastospores can leach through a sand profile using a modified pipette sampling technique. Most studies have used conidia which have hydrophobic properties on their surface, whereas blastospores may differ significantly in their leaching capacity due to their hydrophilic surface properties [35,37]. The effect of drenching the *Ifr* inoculum applied on the surface and the depth the blastospores leached per rate varied significantly in the sand profile. The highest rate of water (1500 mL) allowed some inoculum to leach to the greatest depth in the sand profile; however, it only resulted in 2%–4% mortality of the DRW larvae. This lack of mortality may be directly related to the larvae possibly avoiding contact with the *Ifr* blastospores or if contaminated, removing them by crawling further through the sand as discussed above [34,35,36]. Following application of *M. anisopliae* conidia to the DRW larval cuticle, larvae removed 68%–91% of the conidia during movement through untreated soil at all soil moistures [35]. These authors suggested that DRW larvae removed the fungal conidia prior to germination and penetration during their exposure to the soil. However, in this study, it was not determined how deep in the sand profile the larvae actually burrowed per pot; therefore, the amount of the inoculum that each DRW larva was exposed to was unknown and may have affected the resulting mortality per larva.

Results suggest that an efficient control strategy for DRW may be to apply *Ifr* on plant foliage to manage the adult populations. However, another strategy is still needed to control the beetle larvae that spends the majority of its life time underground feeding on the roots of the tree. As part of an Integrated Pest Management (IPM) program, entomopathogenic fungi are most functional in managing pest populations when incorporated with other biological control agents [30]. Various authors have tested different types of entomopathogenic nematodes used against beetle larvae including the DRW [46,47,48,49]. Parasitic nematodes in the genera *Steinernema* and *Heterorhabditis* have been effective in reducing larval populations by 70%–90% when applied at a rate of 2 billion infective juveniles per acre as a soil drench beneath the tree canopy [49]. However, entomopathogenic nematode field efficacy is often inconsistent and unsatisfactory [50,51], due to various biotic [52,53] and abiotic factors [54,55,56]. Also, although entomopathogenic fungi such as *M. anisopliae* and *B. bassiana* are pathogenic to white grubs [57,58], their performances can be constrained by environmental conditions [59,60].

Recent research has shown that the combined application of fungal entomopathogens and entomopathogenic nematodes may achieve a higher level of control against white grubs. When applied together, they may act independently and cause an additive effect, or interact with each other in a synergistic or antagonistic way [61]. Additive or synergistic interaction has been reported in the combined application of *H. bacteriophora* and *M. anisopliae* MM against the barley chafer grub, *Coptognathus curtipennis* Faimaire [62]. Similar effects were found in the interaction between *M. anisopliae* CLO 53 and *H. megidis* Poinar, Jackson and Klein or *S. glaseri* (Steiner) against the 3rd instar Welsh chafer, *Hoplia philanthus* Füessly under laboratory and greenhouse conditions [63], and between *M. anisopliae* CLO 53 and *H. bacteriophora* in the field [64]. Choo et al. [65] also reported that the combination of *S. carpocap1ae* (Weiser) with *B. brongniartii* (Saccardo) Petch resulted in a significant increase in the mortality of oriental beetle, *Exomala orientalis* (Waterhouse) grubs compared to the application of fungus alone. Most recently the combined application of the fungus *Ifr* CCM 8367 with entomopathogenic nematodes increased the mortality of the Colorado potato beetle larva up to 98.0% compared to either entomopathogen applied alone [66]. Jabbour et al. [67] concluded after investigating entomopathogen biodiversity and its effect on host mortality, that the combined pairings of fungal entomopathogens and entomopathogenic nematodes always produced host mortality that exceeded predictions based on the impact of either entomopathogen alone. Although the combination control treatment of entomopathogenic fungi and entomopathogenic nematodes is promising for other beetle larval pests, this interaction needs to be evaluated using *Ifr* against DRW under greenhouse and various field conditions. However, it is anticipated that additive or synergistic interactions would be achieved from the combined use of the two types of entomopathogens and thus improve the overall efficacy for management of the larval DRW pests.

## 5. Conclusions

Topical spray applications caused 13% and 19% mortality in DRW larvae and adults after 14 days, respectively. When DRW were fed only one *Ifr* treated leaf, 100% mortality occurred 35 days post-treatment. *Ifr* CFUs g^−1^ were isolated 8.5–10.5 cm below the sand surface for the 1000 and 1500 mL drench rates only, resulting in 2%–4% larval mortality. For all the *Ifr* drench treatments, no differences were observed in percent larval mortality and size or the effect of treatments in reducing feeding damage to the plant root biomass compared to the controls. Biological control measures using *Ifr* or other entomopathogenic fungi may provide an additional treatment against DRW adults, while benefits from soil drenching may not significantly increase larval mortality. However, a better means of *Ifr* soil application, or using a higher concentration of inoculum with improved timing of drench treatments during the early springtime when adults oviposit and are susceptible to infection, may improve the efficacy of this fungus for controlling the larval DRW in the soil. Future studies involving the combined application of fungal entomopathogens and entomopathogenic nematodes need to be tested to determine if the overall efficacy for the management of the larval DRW insect pests can be improved.

## Abbreviations

The following abbreviations are used in this manuscript:

DRW	*Diaprepes* root weevil
IPM	Integrated pest management

## References

[1] Woodruff R.E. (1964). A Puerto Rican weevil new to the United States (Coleoptera: Curculionidae). Fla. DPI Entomol. Circ..

[2] Duncan L.W., Rogers M.E., McCoy C.W., Futch S.H., Graham J.H. Florida citrus pest management guide: Citrus root weevils. http://edis.ifas.ufl.edu/cg006.

[3] Rogers S., Graham J.H., McCoy C.W. (1996). Insect-plant interactions: Preliminary studies of *Diaprepes* root weevil injuries and *Phytophthora* infections. Proc. Fla. State Hort. Soc..

[4] Graham J.H., Bright D.B., McCoy C.W. (2003). *Phytophthora-Diaprepes* weevil complex: *Phytophthora* spp. relationship to citrus rootstocks. Plant Dis..

[5] Lapointe S.L., Shapiro J.P. (1999). Effect of soil moisture on development of *Diaprepes abbreviatus* (Coleoptera: Curculionidae). Fla. Entomol..

[6] Puterka G.J. (1999). Fungal pathogens for arthropod pest control in orchard systems: Mycoinsecticidal approach for pear psylla control. Biocontrol.

[7] Subandiyah S., Nikoh N., Sato H., Wagiman F., Tsuyumu S., Fukatsu T. (2000). Isolation and characterization of two entomopathogenic fungi attacking *Diaphorina citri* (Homoptera, Psylloidea) in Indonesia. Mycoscience.

[8] Slininger P., Behle R.W., Jackson M.A., Schisler D.A. (2003). Discovery and development of biological agents to control crop pests. Neotrop. Entomol..

[9] Dolinski C., Lacey L. (2007). Microbial control of arthropod pests of tropical tree fruits. Neotrop. Entomol..

[10] Lacey L.A., Shapiro-Ilan D.I. (2008). Microbial control of insect pests in temperate orchard systems: Potential for incorporation into IPM. Annu. Rev. Entomol..

[11] Guerri-Agullo B., Gomez-Vidal S., Asensio L., Barranco P., Lopez-Llorca L. (2010). Infection of the red palm weevil (*Rhynchophorus ferrugineus*) by entomopathogenic fungus *Beauveria bassiana:* A SEM study. Microsc. Res. Technol..

[12] Vallet-Gely I., Lemaitre B., Boccard F. (2008). Bacterial strategies to overcome insect defenses. Nat. Rev. Microbiol..

[13] Hajek A., Leger R. (1994). Interactions between fungal pathogens and insect hosts. Annu. Rev. Entomol..

[14] Beavers J.B., McCoy C.W., Kanavel R.F., Sutton R.A., Selhime A.G. (1972). Two muscardine fungi pathogenic to *Diaprepes abbreviatus*. Fla. Entomol..

[15] Beavers J.B., Kaplan D.T., McCoy C.W. (1982). Natural enemies of *Diaprepes abbreviatus* larvae in Florida. Proc. Fla. State Hortic. Soc..

[16] Peña J., Giblin-Davis R., Duncan R. (1995). Impact of indigenous *Beauveria bassiana* (Balsamo) Vuillemin on banana weevil and rotten sugarcane weevil (Coleoptera: Curculionidae) populations in banana in Florida. J. Agric. Entomol..

[17] Hussain A., Rizwan-ul-Haq M., Al-Ayedh H., Ahmed S., Al-Jabr A.M. (2015). Effect of *Beauveria bassiana* infection on the feeding performance and antioxidant defence of red palm weevil, *Rhynchophorus ferrugineus*. BioControl.

[18] Lacey L.A., Grzywacz D., Shapiro-Ilan D.I., Frutos R.M., Brownbridge M., Goettel M.S. (2015). Insect pathogens as biological control agents: Back to the future. J. Invertebr. Pathol..

[19] Montemayor C., Avery P.B., Cave R.D. (2016). Infection and mortality of *Microtheca ochroloma* (Coleoptera: Chrysomelidae) by *Isaria fumosorosea* (Hypocreales: Cordycipitaceae) under laboratory conditions. Biocontrol Sci. Technol..

[20] Luangsa-Ard J., Houbraken J., van Doorn T, Hong S.B., Borman A.M., Hywel-Jones N.L., Samson R.A. (2011). *Purpureocillium*, a new genus for the medically important *Paecilomyces lilacinus*. FEMS Microbiol. Lett..

[21] Meyer J., Hoy M.A., Boucias D.G. (2008). Isolation and characterization of an *Isaria fumosorosea* isolate infecting the Asian citrus psyllid in Florida. J. Invertebr. Pathol..

[22] Avery P.B., Hunter W.B., Hall D.G., Jackson M.A., Powell C.A., Rogers M.E. (2009). *Diaphorina citri* (Hemiptera: Psyllidae) infection and dissemination of the entomopathogenic fungus *Isaria fumosorosea* (Hypocreales: Cordycipitaceae) under laboratory conditions. Fla. Entomol..

[23] Hoy M., Singh R., Rogers M.E. (2010). Evaluations of A novel isolate of *Isaria fumosorosea* for control of the Asian citrus psyllid, *Diaphorina citri* (Hemiptera: Psyllidae). Fla. Entomol..

[24] Avery P.B., Wekesa V.W., Hunter W.B., Hall D.G., McKenzie C.L., Osborne L.S., Powell C.A., Rogers M.E. (2011). Effects of the fungus *Isaria fumosorosea* (Hypocreales: Cordycipitaceae) on reduced feeding and mortality of the Asian citrus psyllid, *Diaphorina citri* (Hemiptera: Psyllidae). Biocontrol Sci. Technol..

[25] Stauderman K., Avery P., Aristizabal L., Arthurs S. (2012). Evaluation of *Isaria fumosorosea* (Hypocreales: Cordycipitaceae) for control of the Asian citrus psyllid, *Diaphorina citri* (Hemiptera: Psyllidae). Biocontrol Sci. Technol..

[26] Avery P.B., Pick D.A., Aristizábal L.F., Kerrigan J., Powell C.A., Rogers M.E., Arthurs S.P. (2013). Compatibility of *Isaria fumosorosea* (Hypocreales: Cordycipitaceae) blastospores with agricultural chemicals used for management of the Asian citrus psyllid, *Diaphorina citri* (Hemiptera: Liviidae). Insects.

[27] Ayala-Zermeño M.A., Gallou A., Berlanga-Padilla A.M., Serna-Domínguez M.G., Arredondo-Bernal H.C., Montesinos-Matías R. (2015). Characterisation of entomopathogenic fungi used in the biological control programme of *Diaphorina citri* in Mexico. Biocontrol Sci. Technol..

[28] Casique-Valdés R., Sánchez-Lara B.M., Ek-Mass J., Hernández-Guerra C., Bidochka M., Guízar-Guzmán L., López-Arroyo J., Sánchez-Peña S.R. (2015). Field trial of aqueous and emulsion preparations of entomopathogenic fungi against the Asian citrus psyllid (Hemiptera: Liviidae) in a lime orchard in Mexico. J. Entomol. Sci..

[29] Hunter W.B., Avery P.B., Pick D., Powell C.A. (2011). Broad spectrum potential of *Isaria fumosorosea* on insect pests of citrus. Fla. Entomol..

[30] Shah P.A., Pell J.K. (2003). Entomopathogenic fungi as biological control agents. Appl. Microbiol. Biotechnol..

[31] Jackson M.A., Erhan S., Poprawski T.J. (2006). Influence of formulation additives on the desiccation tolerance and storage stability of blastospores of the entomopathogenic fungus *Paecilomyces fumosoroseus* (Deuteromycotina: Hyphomycetes). Biocontrol Sci. Technol..

[32] McCoy C.W., Stuart R.J., Duncan L.W., Shapiro-Ilan D.I., Lacey L.A., Kaya H.K. (2007). Application and evaluation of entomopathogens for citrus pest control. Field Manual of Techniques in Invertebrate Pathology.

[33] Goettel M.S., Inglis G.D., Lacey L.A. (1997). Fungi: Hyphomycetes. Manual of Techniques in Insect Pathology.

[34] Yaginuma D., Hiromori H., Hatsukade M. (2006). Friction-associated conidial detachment of the entomopathogenic fungus *Beauveria amorpha* from the cuticle of a scarab grub, *Anomala cuprea*, in the soil. Appl. Entomol. Zool..

[35] Quintela E.D., McCoy C.W. (1998). Synergistic effect of imidacloprid and two entomopathogenic fungi on the behavior and survival of larvae of *Diaprepes abbreviatus* (Coleoptera: Curculionidae) in soil. J. Econ. Entomol..

[36] Gillett-Kaufman J.L., Kimbrough J.W. (2009). A modified method to visualize infection sites of spores of the entomopathogen *Beauveria bassiana* (Deuteromycotina: Hyphomycetes) on the exoskeleton of citrus root weevil *Diaprepes abbreviatus* (Coleoptera: Curculionidae) adults. Fla. Entomol..

[37] Vega F.E., Meyling N.V., Luangsa-ard J.J., Blackwell M., Kaya H.K., Vega F.E. (2012). Fungal entomopathogens. Insect Pathology.

[38] Woods S.P., Grula E.A. (1984). Utilizable surface nutrients on *Heliothis zea* available for growth of *Beauveria bassiana*. J. Invertebr. Pathol..

[39] Sosa-Gomez D.R., Boucias D.G., Nation J.L. (1997). Attachment of *Metarhizium anisopliae* to the southern green stink bug *Nezara viridula* cuticle and fungistatic effect of cuticular lipids and aldehydes. J. Invertebr. Pathol..

[40] Hunt D.W.A., Borden J.H., Rahe J.E., Whitney H.S. (1984). Nutrient-mediated germination of *Beauveria bassiana* conidia on the integument of the bark beetle *Dendroctonus ponderosae* (Coleoptera: Scolytidae). J. Invertebr. Pathol..

[41] Oritz-Urquiza A., Keyhani N.O. (2013). Action on the surface: Entomopathogenic fungi versus the insect cuticle. Insects.

[42] Kaya H.K., Vega F.E., Kaya H.K., Vega F.E. (2012). Scope and basic principles of insect pathology. Insect Pathology.

[43] Fargues J., Delmas J.C., Lebrun R.A. (1994). Leaf consumption by larvae of the Colorado potato beetle (Coleoptera: Chrysomelidae) infected with the entomopathogen, *Beauveria bassiana*. J. Econ. Entomol..

[44] Ekesi S. (2001). Pathogenicity and antifeedant activity of entomopathogenic hyphomycetes to the cowpea leaf beetle, *Ootheca mutabilis* Shalberg. Int. J. Trop. Insect Sci..

[45] Gámez Herrera C., Niño A.A., Avery P.B., Cave R.D. (2016). Efecto del hongo *Isaria fumosorosea* Wize sobre la supervivencia y el consumo de los adultos del escarabajo demargen amarillo, *Microtheca ochroloma* Stål (Coleoptera: Chrysomelidae). CEIBA.

[46] Duncan L.W., McCoy C.W. (1996). Vertical distribution in soil, persistence, and efficacy against citrus root weevil (Coleoptera: Curculionidae) of two species of entomogenous nematodes (Rhabditida: Steinernematidae: Heterorhabditidae). Environ. Entomol..

[47] Koppenhöfer A.M., Fuzy E.M., Crocker R.L., Gelernter W.D., Polavarapu S. (2004). Pathogenicity of *Steinernema scarabaei*, *Heterorhabditis bacteriophora* and *S. glaseri* to twelve white grub species. Biocontrol Sci. Technol..

[48] Koppenhöfer A.M., Grewal P.S., Fuzy E.M. (2006). Virulence of the entomopathogenic nematodes *Heterorhabditis bacteriophora*, *H. zealandica*, and *Steinernema scarabaei* against five white grub species (Coleoptera: Scarabaeidae) of economic importance in turfgrass in North America. Biol. Control..

[49] McCoy C.W., Duncan L.W., Quintela E.D. (1996). A review of IPM strategies for citrus root weevils with emphasis on microbial control. Proc. Int. Soc. Citric..

[50] Georgis R., Gaugler R. (1991). Predictability in biological control using entomopathogenic nematodes. J. Econ. Entomol..

[51] Klein M.G., Bedding R., Akhurst R., Kaya H.K. (1993). Biological control of scarabs with entomopathogenic nematodes. Nematodes and The Biological Control of Insect Pests.

[52] Kaya H.K., Gaugler R. (2002). Natural enemies and other antagonists. Entomopathogenic Nematology.

[53] Kaya H.K., Koppenhöfer A.M. (1996). Effects of microbial and other antagonistic organisms and competition on entomopathogenic nematodes. Biocontrol Sci. Technol..

[54] Kaya H.K., Gaugler R., Kaya H.K. (1990). Soil ecology. Entomopathogenic Nematodes in Biological Control.

[55] Smits P.H. (1996). Post-application persistence of entomopathogenic nematodes. Biocontrol Sci. Technol..

[56] Glazer I., Gaugler R. (2002). Survival biology. Entomopathogenic Nematology.

[57] Glare T.R., Placet C., Nelson T.L., Reay S.D. (2002). Potential of *Beauveria* and *Metarhizium* as control agents of pinhole borers (*Platypus* spp.). NZ Plant Protect. J..

[58] Mohammadyani M., Karimi J., Taheri P., Sadeghi H., Zare R. (2016). Entomopathogenic fungi as promising biocontrol agents for the rosaceous longhorn beetle, *Osphranteria coerulescens*. BioControl.

[59] Wraight S.P., Inglis G.D., Goettel M.S., Lacey L.A., Kaya H.K. (2007). Fungi. Field Manual of Techniques in Invertebrate Pathology.

[60] Hasan S. (2014). Entomopathogenic fungi as potent agents of biological control. IJETR.

[61] Jaques R., Morris O.N., Burges H.D. (1981). Compatibility of pathogens with other methods of pest control and with different crops. Microbial Control of Pests and Plant Disease 1970–1980.

[62] Anbesse S.A., Adge B.J., Gebru W.M. (2008). Laboratory screening for virulent entomopathogenic nematodes (*Heterorhabditis bacteriophora* and *Steinernema yirgalemense*) and fungi (*Metarhizium anisopliae* and *Beauveria bassiana*) and assessment of possible synergistic effects of combined use against grubs of the barley chafer *Coptognathus curtipennis*. Nematology.

[63] Ansari M.A., Tirry L., Moens M. (2004). Interaction between *Metarhizium anisopliae* CLO 53 and entomopathogenic nematodes for the control of *Hoplia philanthus*. Biol. Control.

[64] Ansari M.A., Shah F.A., Tirry L., Moens M. (2006). Field trials against *Hoplia philanthus* (Coleoptera: Scarabaeidae) with a combination of an entomopathogenic nematode and the fungus *Metarhizium anisopliae* CLO 53. Biol. Control.

[65] Choo H.Y., Kaya H.K., Huh J., Lee D.W., Kim H.H., Lee S.M., Choo Y.M. (2002). Entomopathogenic nematodes (*Steinernema* spp. and *Heterorhabditis bacteriophora*) and a fungus *Beauveria brongniartii* for biological control of the white grubs, *Ectinohoplia rufipes* and *Exomala orientalis*, in Korean golf courses. Biol. Control.

[66] Hussein H.M., Skoková Habuštová O., Půža V., Zemek R. (2016). Laboratory evaluation of *Isaria fumosorosea* CCM 8367 and *Steinernema feltiae* Ustinov against immature stages of the Colorado potato beetle. PLoS ONE.

[67] Jabbour R., Crowder D.W., Aultman E.A., Snyder W.E. (2011). Entomopathogen biodiversity increases host mortality. Biol. Control.

